# Off-Stoichiometry Thiol-Ene Surface Functionalization: Example with Gold Nanoparticles

**DOI:** 10.3390/ma17246135

**Published:** 2024-12-15

**Authors:** Rihards Ruska, Anatolijs Sarakovskis, Edmunds Zutis, Gunita Paidere, Igor Vozny, Janis Cipa, Jevgenijs Gabrusenoks, Toms Freimanis, Raivis Zalubovskis, Andris Anspoks

**Affiliations:** 1 Institute of Solid State Physics, University of Latvia, 8 Kengaraga Street, LV-1063 Riga, Latvia; edmunds.zutis@cfi.lu.lv (E.Z.); gunita.paidere@cfi.lu.lv (G.P.); janis.cipa@cfi.lu.lv (J.C.); jevgenijs.gabrusenoks@cfi.lu.lv (J.G.); andris.anspoks@cfi.lu.lv (A.A.); 2 Latvian Institute of Organic Synthesis, 21 Aizkraukles Street, LV-1006 Riga, Latvia; igor_vozny@osi.lv (I.V.); freimanis.toms@outlook.lv (T.F.); raivis@osi.lv (R.Z.); 3 Institute of Chemistry and Chemical Technology, Faculty of Natural Sciences and Technology, Riga Technical University, P. Valdena iela 3, LV-1048 Riga, Latvia

**Keywords:** OSTE, thiol groups, linkers, gold nanoparticles, Raman spectroscopy, XPS

## Abstract

Surface modification is essential in microfluidic applications due to the inherent hydrophobicity of polymers, which can lead to biofouling and reagent denaturation. Despite the development, challenges such as hydrophobic molecule absorption and limitations in scaling are still present. Off-stoichiometry thiol-ene (OSTE) materials have emerged as a promising alternative, offering advantages like rapid prototyping, minimal hydrophobic absorption, and customizable surface chemistries. While the thiol-ene polymerization mechanism is well understood, the fundamental understanding of thiol group binding on OSTE surfaces remains limited. Existing techniques to analyze surface groups lack the capability to confirm the stable presence of thiol groups on the surface. In our study, using Raman and X-ray photoelectron spectroscopy techniques, we investigated a potential method for enhancing the surface properties of OSTE polymer—the attachment of novel linkers to the surface. We have demonstrated our synthesized compound efficiency by binding gold nanoparticles to the OSTE surface. Our findings indicate that chemical reactions involving double bonds with the material surface hold the most potential for effective surface modification for gold binding.

## 1. Introduction

Surface modification is often crucial for microfluidic applications as polymers are usually hydrophobic. This hydrophobicity can lead to biofouling or high surface adsorption, which can lead to the denaturation of reagents [1]. To address these issues, multiple protocols for surface modification have been developed, including plasma treatment [2], UV irradiation [3], coatings against biofouling [4], and others [5]. Nevertheless, considerable research has been dedicated to protocol development for polydimethylsiloxane (PDMS) as it is the preferred material in many microfluidics devices [6].

PDMS is commonly used due to its affordability, ease of handling, optical transparency, and adhesion to a variety of substrates, as well as its gas permeability [7]. Although PDMS surfaces can be modified using various techniques, it presents challenges in achieving reliable device integration and often exhibits limitations such as high hydrophobic molecule absorption and difficulties in scaling up production [8].

To address these issues and facilitate easier fabrication, off-stoichiometry thiol-ene (OSTE) materials have gained increasing attention within the microfluidics community. OSTE materials offer a range of favorable properties, including rapid prototyping, minimal hydrophobic molecule absorption, and customizable surface chemistries [8]. This flexibility makes OSTE an attractive alternative for being used in applications such as nanoliter well arrays for liquid storage [9], synthetic paper for immunoassays [10], plasma separation devices [11], and extracellular vesicle (EV) extraction systems [12].

The OSTE material mechanisms are well understood and follow the thiol reaction first described in 1938 [13]. The main mechanism of the thiol-ene polymerization process consists of three main phases. In the initiation phase, a UV-sensitive initiator is irradiated to form a radical, which extracts the hydrogen from a thiol monomer. The formed thiyl-radical is then able to propagate (propagation phase) or terminate (termination phase). During the propagation phase, the chain transfer propagation steps or polymerization take place [13,14]. The reaction in OSTE materials is facilitated by using two components—thiol and allyl monomers—allowing the material’s surface properties to be tuned by adjusting the concentration of each component. This distinctive feature enables control over the surface chemistry, allowing for transitions between an allyl-excess state, a stoichiometric balance, or a thiol-excess configuration [8].

Despite knowledge of the materials’ bulk, our fundamental understanding of the thiol group binding on the material’s surface remains limited. The surface properties, including wettability [15] and long-term stability [16], have been previously explored. However, existing techniques used to determine surface groups, including optical methods [8,17] and Ellman’s procedure using pentaerythritol tetrakis-(3-mercaptopropionate), 1,3,5-triallyl-1,3,5-triazine-2,4,6(1H,3H,5H)-trione, and 5,5′-dithiobis(2-nitrobenzoic acid) (DTNB) to detect thiol groups [15,16,18], are unable to confirm whether these groups are stable and present on the surface or are located within a few nanometers below it. This limitation has resulted in inconsistencies in the reported protocols and challenges in thiol group functionalization without UV exposure [17] or surface dissolution [15].

To address these issues, we developed novel linkers with a terminal double bond to facilitate more precise surface functionalization, providing an alternative strategy for achieving stable attachment and enhancing the versatility of OSTE materials in microfluidic applications. In addition, we employ Raman and X-ray photoelectron spectroscopy (XPS) to investigate the binding of gold nanoparticles to the OSTE surface.

## 2. Materials and Methods

### 2.1. Mold Preparation

**Figure 1:**
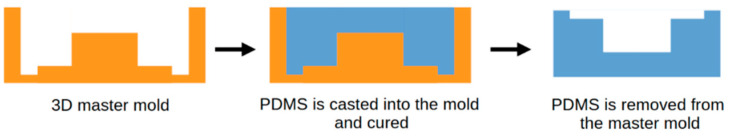
PDMS mold preparation.

For the fabrication of devices, an ultraviolet light liquid crystal display (UV LCD) stereolithography 3D printer (Zortrax Inkspire, Olsztyn, Poland) was used to print a polymer resin (Zortrax Inkspire white/ivory BASIC resin) mold consisting of 5 linked cylinders (shaped like ice-hockey pucks) with a diameter of 10 mm and a depth of 1.50 mm. The double-negative mold was further used to fabricate a negative PDMS (Sylgard TM 184 Silicone Elastomer, Dow, Midland, MI, USA) mold. The PDMS components were combined (crosslinker–base ratio 1:10 (*w*/*w*)) and mixed in a planetary mixer for 1 min at 500 RPM. The mixture was then poured into the 3D-printed mold and degassed in a vacuum chamber to remove air bubbles in the material. The mold’s top part was covered with a Teflon sheet, placed in a custom-made holder, and cured for 3 h at 60 °C. After curing, the silicone mold was removed from the 3D mold, rinsed with 2-propanol (99.8%), and blow-dried under nitrogen flow. The manufacturing process is shown in Figure 1. This protocol has been successfully implemented in a previous study by our group [19].

### 2.2. Fabrication of OSTE Samples

Commercially obtained OSTE components (Ostemer 220 Litho, Reagent A and Reagent B, Mercene Labs, Stockholm, Sweden) were prepared and mixed in a planetary mixer for 5 min at 750 RPM followed by a defoaming step for 5 min at 750 RPM. The mixed material was transferred to a conical centrifuge tube (Falcon^®^, Corning Inc., Corning, NY, USA) and degassed for 0.5–1 h in a vacuum chamber.

**Figure 2:**
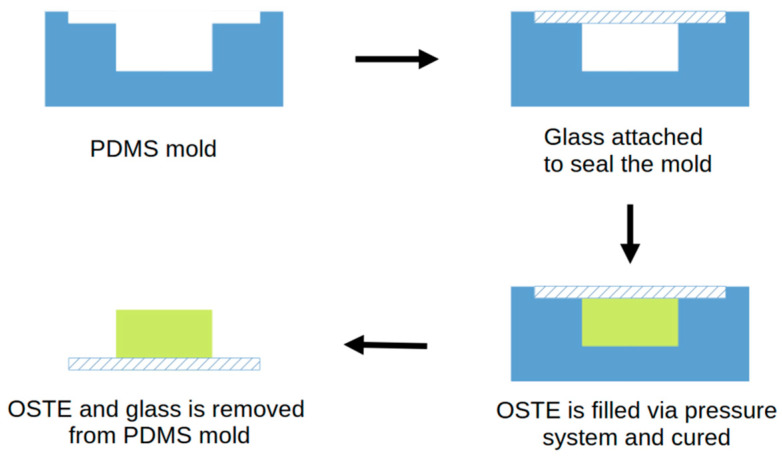
OSTE sample preparation.

Further, the tube was attached to a microfluidic pressure system (OB1 MK3+, Elveflow, Paris, France). A glass slide was placed onto the structured side of the PDMS mold and placed in a custom-made jig, which provided a sealed environment. The assembly was attached to the pressure system and the mold’s cavities were then filled with OSTE polymer by using a 1000 mbar pressure. The manufacturing process is shown in Figure 2 [19].

After filling, the mold was disconnected from the pressure system and immediately cured under UV light (Mask aligner, model MA6, Suss, Garching, Germany) with a dose of 300 mJ/cm^2^ using ND33 and I-line filters to ensure a lower light intensity for better reaction dynamics. The sample consisting of the glass slide with interconnected OSTE cylinders was then removed from the mold. The cylinders were then separated by cutting off the excess OSTE polymer.

### 2.3. Synthesis of the Linker Compounds

**Figure 3:**
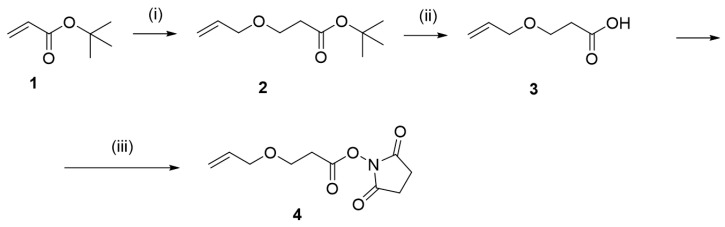
Reagents and conditions: (i) allyl alcohol, Cs_2_CO_3_, DMF, 40 °C, 16 h (ii) TFA, CH_2_Cl_2_, 40 °C, 16 h; (iii) *N*-hydroxysuccinimide, EDC*HCl, DMAP, CH_2_Cl_2_, 16 h.

**Figure 4:**
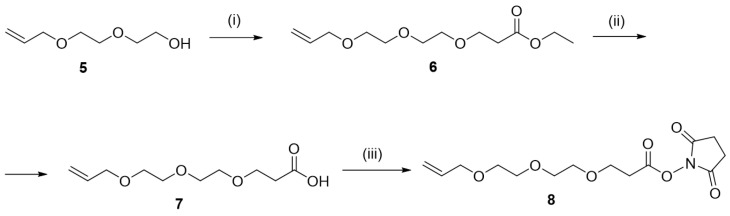
Reagents and conditions: (i) ethyl acrylate, Na (0.3 equiv.), THF, rt, 16 h; (ii) KOH, MeOH, H_2_O, rt, 16 h; (iii) *N*-hydroxysuccinimide, EDC*HCl, DMAP, CH_2_Cl_2_, 0 °C to rt, 16 h.

The syntheses of the linkers are depicted in Figure 3 and Figure 4. In detail, the synthesis of Linker 1 (**4**) started from commercially available t-butyl acrylate (**1**). Treatments of 1 with allyl alcohol in the presence of Cs_2_CO_3_ provided adduct **2**. Ester **2** was hydrolyzed by treatment with trifluoracetic acid (TFA), thus providing acid **3**. The reaction of **3** with *N*-hydroxysuccinimide gave the desired Linker 1.

The synthesis of Linker 2 (**8**) started with alcohol **5**, which was treated with ethyl acrylate (Figure 4). It is important to note that the patent literature indicates that 0.3 equiv. of sodium should be used in the reaction [20]. Indeed, we found out that using 1 equiv. of sodium leads to an inseparable mixture of byproducts, where the desired **6** was a minor one. However, using the patent-described conditions provided nearly pure compound **6**, which was further hydrolyzed under basic conditions to acid **7**. The treatment of acid **7** by *N*-hydroxysuccinimide provided the desired Linker 2.

#### 2.3.1. Tert-Butyl 3-(Allyloxy)propanoate (2)

t-Butyl acrylate (**1**) (1.47 mL, 10.0 mmol, 1 equiv.) was dissolved in dry DMF (6 mL) and then cesium carbonate (3.24 g, 10.0 mmol, 1 equiv.) was added and stirred at 0 °C for 10 min. Then, allyl alcohol (0.61 mL, 10.0 mmol, 1 equiv.) was dissolved in dry DMF (4 mL) and cooled to 0 °C, and this solution was added dropwise to the solution of tert-butyl acrylate. The reaction was stirred for 1 h in an ice bath and then at 40 °C for 18 h. Brine (15 mL) was added and extracted with diethyl ether (3 × 20 mL). Combined extract was washed with brine (20 mL) and water (3 × 20 mL) and dried over Na_2_SO_4_ and evaporated. The crude material was used directly in the next step.

#### 2.3.2. 3-(Allyloxy)propanoic Acid (3)

Crude **2** from the last step was dissolved in dry DCM (20 mL) and then trifluoroacetic acid (1.30 mL, 16.6 mmol, 2.5 equiv.) was added. The reaction was stirred at 40 °C for 18 h. The volatiles were evaporated and then traces of TFA were evaporated with the addition of 5 mL of toluene twice. The residue was purified on silica gel column PE/EtOAc (5:1). Product **3** (810 mg, 70%) was obtained as a colorless oil.

#### 2.3.3. 2,5-Dioxopyrrolidin-1-yl 3-(Allyloxy)propanoate **(4)**

Allyl propionic acid (**3**) (500 mg, 6.22 mmol, 1 equiv.) was dissolved in dry DCM (20 mL), and then *N*-hydroxysuccinimide (1.08 g, 9.34 mmol, 1.5 equiv.) was added. Then, 1-ethyl-3-(3-dimethylaminopropyl) carbodiimide (1.78 g, 9.34 mmol, 1.5 equiv.) and 4-dimethylaminopyridine (1.52 g, 12.45 mmol, 2 equiv.) were added and the reaction mixture was stirred at room temperature for 18 h. Volatiles were evaporated in vacuum, and the residue was purified on silica gel column DCM/MeOH (10:1). Compound **4** (270 mg, 19%) was obtained as a colorless oil.

^1^H NMR (300 MHz, CDCl_3_) δ 5.97–5.83 (m, 1H), 5.33–5.16 (m, 2H), 4.02 (dt, *J* = 5.6, 1.4 Hz, 2H), 3.79 (t, *J* = 6.4 Hz, 2H), 2.89 (t, *J* = 6.4 Hz, 2H), 2.83 (s, 4H).
^13^C NMR (150 MHz, CDCl_3_) δ 169.1, 166.8, 134.4, 117.6, 72.3, 64.6, 32.3, 25.7.
HRMS (ESI) [M + Na]^+^: m/z calcd for (C_10_H_13_NO_5_Na) 250.0691. Found 250.0699.

#### 2.3.4. Ethyl 3-(2-(2-(Allyloxy)ethoxy)ethoxy)propanoate **(6)**

To a stirred solution of 2-(2-(allyloxy)ethoxy)ethan-1-ol (**5**) (680 mg, 4.65 mmol, 2.2 equiv.) in dry THF (20 mL), Na (15 mg, 0.63 mmol, 0.3 equiv.) was added at room temperature. NOTE: using a larger amount of sodium resulted in the formation of an inseparable mixture of various products. The reaction was then stirred at room temperature for 1 h (until the Na chunks disappeared). A solution of ethyl acrylate (212 mg, 2.11 mmol, 1.0 equiv.) in dry THF (1 mL) was then added. The mixture was stirred at rt overnight. The reaction was quenched with a few drops of glacial acetic acid. Volatiles were evaporated in vacuum. The product was extracted with CHCl_3_ (50 mL). The organic layer was dried over Na_2_SO_4_ and solvent was evaporated under vacuum to provide crude ester **6**, which was used in the next step without additional purification.

#### 2.3.5. 3-(2-(2-(Allyloxy)ethoxy)ethoxy)propanoic acid **(7)**

Ester **6** was diluted with a 1/1 (*v*/*v*) mixture of MeOH (20 mL) and 2.5 M KOH aq. (20 mL). The reaction was vigorously stirred at room temperature overnight and then extracted with CHCl_3_. The aqueous layer was carefully acidified with conc. HCl and then extracted with CHCl_3_ (50 mL). The organic layer was dried over Na_2_SO_4_ and the solvent was evaporated under vacuum to provide crude acid **7** (200 mg, 43%), which was used in the next step without purification.

#### 2.3.6. 2,5-Dioxopyrrolidin-1-yl 3-(2-(2-(Allyloxy)ethoxy)ethoxy)propanoate **(8)**

To ice-cold solution of 3-(2-(2-(allyloxy)ethoxy)ethoxy)propanoic acid (**7**) (120 mg, 0.80 mmol, 1 equiv.) in dry CH_2_Cl_2_ (25 mL) *N*-hydroxysuccinimide (138 mg, 1.20 mmol, 1.5 equiv.), DMAP (195 mg, 1.60 mmol, 2 equiv.) and EDC*HCl (230 mg, 1.20 mmol, 1.5 equiv.) were added. The mixture was stirred in a melting ice bath overnight. Extra CH_2_Cl_2_ (70 mL) was added, and the mixture was washed with 1M HCl (10 mL) and sat. NaHCO_3_ (20 mL). The solvent was evaporated in a vacuum. The residue was purified on silica gel column CH_2_Cl_2_/MeOH (19:1). Compound **8** (47 mg, 19%) was isolated as light pink oil.

^1^H NMR (400 MHz, CDCl_3_) δ 5.90 (ddt, *J* = 17.3, 10.4, 5.7 Hz, 1H), 5.26 (dq, *J* = 17.3, 1.6 Hz, 1H), 5.21–5.12 (m, 1H), 4.01 (dt, *J* = 5.7, 1.4 Hz, 2H), 3.84 (t, *J* = 6.5 Hz, 2H), 3.66–3.63 (m, 6H), 3.61–3.57 (m, 2H), 2.89 (t, *J* = 6.5 Hz, 2H), 2.82 (s, 4H).
^13^C NMR (101 MHz, CDCl_3_) δ 169.1, 166.8, 134.9, 117.2, 72.3, 70.8, 70.8, 70.7, 69.5, 65.8, 32.3, 25.7.
HRMS (ESI) [M + Na]^+^: *m*/*z* calcd for (C_14_H_21_NO_7_Na) 338.1216. Found 338.1229.

### 2.4. Functionalization of OSTE Surface

To functionalize the surface of the OSTE cylinders, 10 mM solutions of linker compounds **4** (Linker 1) and **8** (Linker 2) were prepared in acetone (Purris p.a, Honeywell, Charlotte, NC, USA). A total of 100 μL of each linker solution was deposited onto each OSTE cylinder using a pipette (Eppendorf Research plus 1-channel, 10–100 μL, Eppendorf, Hamburg, Germany). For the control measurements, 100 μL of pure acetone was used. The samples were then exposed under UV light with a dose of 4000 mJ/cm^2^ using ND33 and I-line filters to ensure the covalent attachment of the linkers to the OSTE surface. After exposure, samples were rinsed under a deionized water stream and blow-dried under nitrogen flow.

To introduce the –SH groups to the surface, samples were further washed with 2-propanol (≥99.5%, Merck, Darmstadt, Germany). Then, 100 μL of concentrated 2-aminoethane-1-thiol (99.0%, Fluorochem, Hadfield, UK) solution in degassed water was applied on top of the OSTE sample surface using the aforementioned pipette and incubated for 2 h. During the incubation step, samples were stored in a nitrogen-filled Petri dish. To further functionalize OSTE with gold nanoparticles, the samples were rapidly rinsed under a deionized water stream and similarly covered with 100 μL of commercially available PBS-stabilized gold nanoparticles (5 nm, 0.01 mM, 99%, Thermo Fisher Scientific, Waltham, MA, USA) for 2 h. The time interval between the start of rinsing and the gold nanoparticle application did not exceed 10 s. During incubation, step samples were kept in air-filled Petri dishes. After that, the samples were thoroughly rinsed under a deionized water stream and dried in air at room temperature for further analyses.

### 2.5. Spectroscopic Measurements

OSTE samples were spectroscopically studied using the standard XPS and Raman methods. Raman measurements were performed for the spectral region 500–4000 cm^−1^ using a confocal triple monochromator Raman system (TriVista CRS, S&I Spectroscopy & Imaging, Warstein, Germany). Excitation of the samples was achieved using a 532 nm laser. XPS measurements were performed using the Thermo Fisher ESCALAB Xi measurement system. The measured spectral data were processed and illustrated in the program Origin Pro 2024.

## 3. Results and Discussion

### 3.1. Study of OSTE Polymerization Process

To gain an understanding of the OSTE properties, several mixtures of components A and B were prepared and irradiated with 100 mJ of UV light. The resultant samples were tested for their shape retention properties and relative 2575 cm^−1^ (to S-H bond stretch) and 1645 cm^−1^ (C=C bond stretch) band intensities [8,21,22].

**Figure 5:**
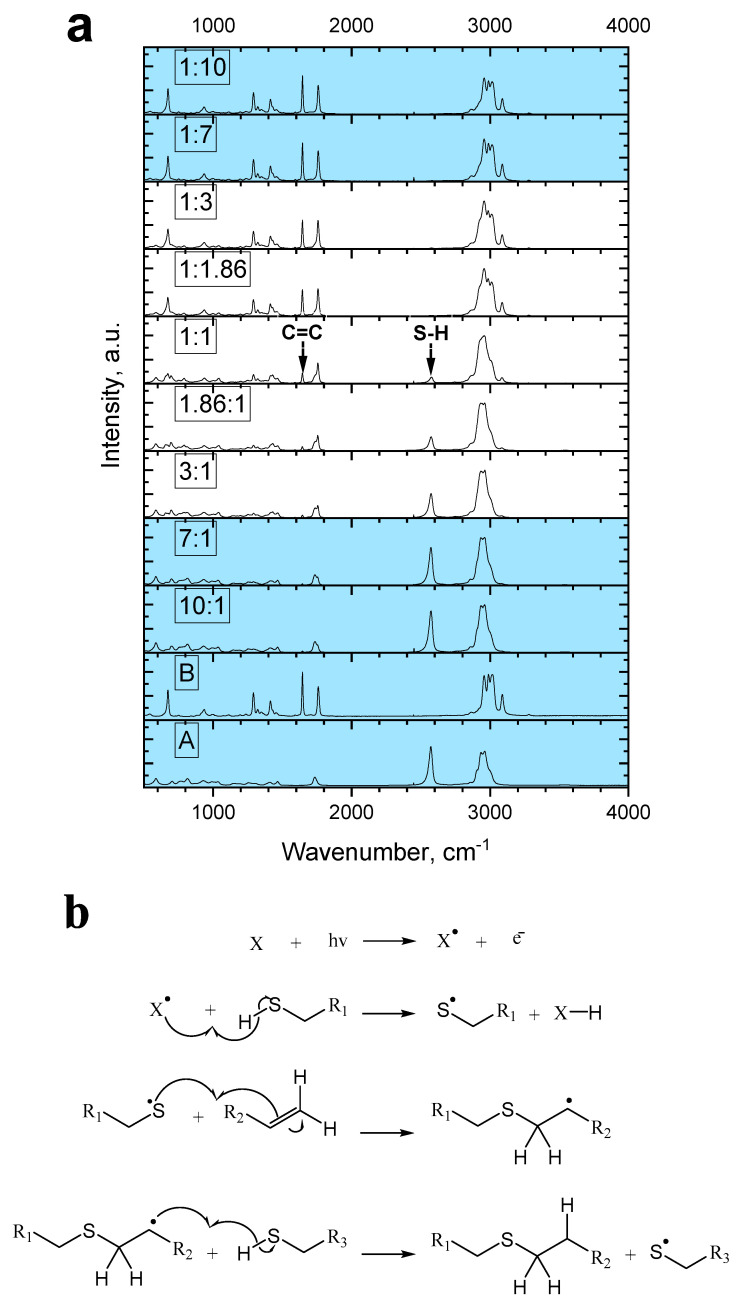
(**a**) Raman measurement results for various OSTE component A:B ratios. Blue coloring of spectra indicates that produced samples were liquid. (**b**) Mechanism of OSTE polymerization (propagation phase) reaction between –SH and C=C functional groups, where X is photoinitiator.

Samples of A:B reagent mixtures within the interval of 1:3 to 3:1 did solidify, but samples outside that window did not and thus were not viable for further surface studies. Raman analysis showed that samples with a higher proportion of reagent A showed an increased count of S-H bonds. On the other hand, samples with a higher reagent B concentration exhibited higher C=C group presence (Figure 5a).

It was also observed that by changing the reagent ratios, the S-H and C=C band relative intensities changed as well. Moreover, reagent ratios close to 1:1 yielded a product with a low presence of both –SH and C=C groups. This means that the polymerization reaction involved these functional groups (Figure 5b). This finding is consistent with the previously published results [8].

#### Binding of Gold Nanoparticles

**Figure 6:**
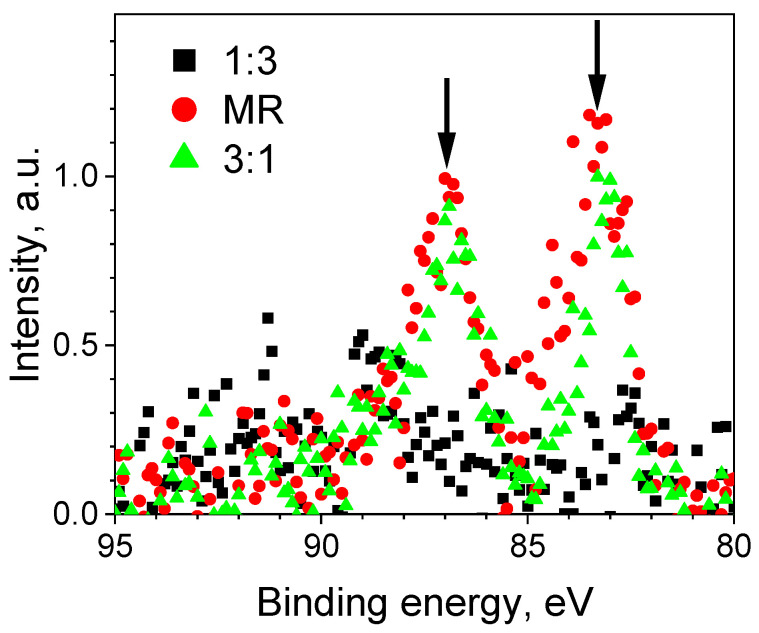
XPS measurement results of Au4f bands (indicated by arrows) on gold-nanoparticle-treated, unfunctionalized OSTE samples (A and B reagent ratios—ratios 3:1, MR, and 1:3).

Since the Raman measurements showed that OSTE contains a large number of –SH groups, it was expected that gold nanoparticles would bind to the OSTE surface. To test this, several OSTE samples (ratios 3:1, 1.86:1 (manufacturer recommended (MR)), and 1:3) were coated in gold nanoparticle solutions and tested for the presence of gold on the OSTE surface using the XPS methods. The results showed that comparably low amounts of gold are present on the 3:1 and MR samples, but no gold was present on the surface of the 1:3 sample (Figure 6). This can be explained by the Raman data, which suggest that while the 3:1 and MR samples contain a notable amount of –SH groups, the 1:3 sample holds a negligible amount of them.

**Figure 7:**
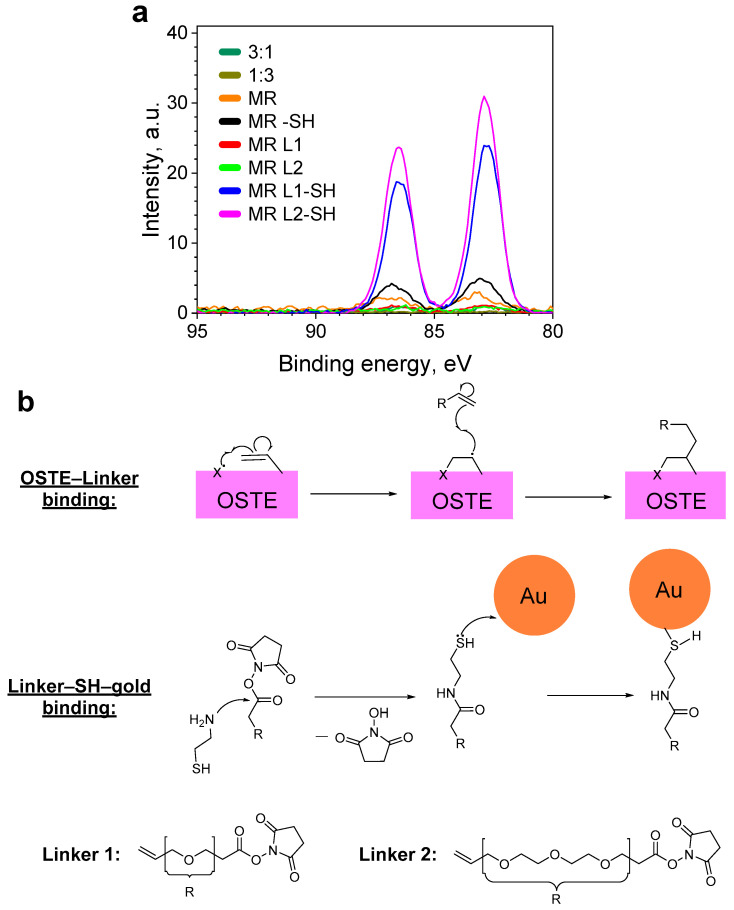
(**a**) XPS measurement results of Au4f bands on gold-treated, unfunctionalized OSTE samples (MR, 3:1, and 1:3 indicate the reagent ratio in OSTE, –SH—thioethanolamine treatment, but L1 and L2—linker treatment; the results are illustrated with corresponding standard deviation intervals (shaded area, *p* = 0.10). (**b**) Schematic reaction mechanisms of linker attachment to the OSTE and gold attachment to the linker-functionalized samples (X is photoinitiator) [24].

Nevertheless, the amounts of adsorbed gold were quite low, as suggested by the noisy XPS spectra. It is known that thiol (–SH) groups in the presence of oxygen can react to form disulfide (-S-S-) groups and liberate water molecules [23]. The resulting disulfide groups are less reactive and cannot bind the gold nanoparticles as effectively as the thiol groups. To test if the OSTE surface could have gone through a surface –SH group oxidation, we prepared functionalized OSTE samples, as detailed in the experimental section: the OSTE surface was coated with Linker 1 or Linker 2 and then treated with 2-aminoethane-1-thiol (thioethanolamine) solution followed by gold nanoparticle solution. The XPS data for the prepared samples are depicted in Figure 7a. It was observed that most samples exhibited the presence of some gold on the surface. This could be explained by both the presence of some –SH groups on the surface as well as gold nanoparticles being physiosorbed onto the OSTE surface/caught in pores. Treating the surface with thioethanolamine solution appeared to increase the amount of bound gold. This could be attributed to the incomplete removal of thioethanolamine during the rinsing step, which increased the available –SH group count for gold nanoparticle binding.

If we look at the results of the linker-treated samples, we can see a notable increase in the amount of attached gold nanoparticles. If compared to the thioethanolamine sample (MR–SH), the increase with Linker 1 is ~5-fold (MR L1–SH), but Linker 2 is ~6-fold (MR L2–SH). If we compare the linker results to the thioethanolamine untreated samples (MR, 3:1), the increase is ~24- and ~30-fold, respectively.

Our XPS and Raman results show that the –SH groups on the surface of the thiol-rich OSTE samples can become inactive. We speculate that this happens due to –SH group oxidation in air and the formation of disulfide groups. Nevertheless, the –SH groups can be reintroduced to the surface of OSTE by functionalizing it with linkers and thioethanolamine. Since –SH group inactivation reduces their activity in radical reactions, we speculate that linker attaches to the unreacted C=C groups, which are more easily activated. In our case, more homogenous results were achieved by using Linker 1, while Linker 2 offered a higher potential for gold nanoparticle binding.

Other benefits that we see in using linkers in OSTE functionalization are their stability, ease of application, and versatility. For one, the time between the linker coating and the following thioethanolamine treatment was 6 h. This time interval was similar to the time between untreated OSTE production and its coating in gold nanoparticle solution. It means that while the –SH groups become inactive after 6-hour exposure to ambient air, the linker samples did not lose their activity. Secondly, our linkers are easy to apply to the OSTE surface—no additional mechanical/chemical treatment was needed after they were attached to the surface. And lastly, if we look at the reaction mechanism for linker–thioethanolamine–gold nanoparticle treatment (Figure 7b), we can see that our linkers could not only be employed in binding –SH-reactive species but also –NH_2_-reactive ones.

## 4. Conclusions

A systemic study of OSTE polymer properties and possibilities for its gold nanoparticle binding enhancement using linkers was performed. Our main findings are as follows:

1. OSTE–SH and terminal C=C group ratio can be varied by changing the ratio of components A and B, used as precursors to OSTE. The higher the A reagent concentration, the higher the –SH group presence. On the other hand, a higher B reagent concentration leads to a higher C=C group presence. Changing the ratios can also affect the visual appearance (liquid or solid) of the resultant samples. Solid samples were produced in the A:B ratio range from 3:1 to 1:3.
2. Unfunctionalized OSTE polymer samples have insufficient binding with gold nanoparticles. Nevertheless, the binding can be significantly improved by employing –NH_2_-reactive linkers. By treating the linker-functionalized samples with thioethanolamine, surface active –SH groups can be introduced to the OSTE polymer surface, thus enabling gold nanoparticle binding.
3. Our reported linkers, in theory, could be used not only for the attachment of substances that react with –SH groups but also with –NH_2_ groups. This property could be employed in attaching various proteins to the OSTE surface as they all contain at least one terminal –NH_2_ group. By changing the terminal groups of our linkers, other molecule binding could theoretically also be achieved.

## Data Availability

The datasets generated and/or analyzed during the current study are not publicly available because the data are part of an ongoing study, but are available from the corresponding author upon reasonable request.
